# Effective Communication Supported by an App for Pregnant Women: Quantitative Longitudinal Study

**DOI:** 10.2196/48218

**Published:** 2024-04-26

**Authors:** Lukas Kötting, Vinayak Anand-Kumar, Franziska Maria Keller, Nils Tobias Henschel, Sonia Lippke

**Affiliations:** 1 Psychology and Methods School of Business, Social & Decision Sciences Constructor University Bremen gGmbH Bremen Germany; 2 Klinikum Bremerhaven Reinkenheide gGmbH Bremerhaven Germany

**Keywords:** clinical care, health action process approach, HAPA, intention, communication behavior, patient safety, patient education, internet intervention, dropout, digital health, behavior change, prediction, obstetric, pregnant women, pregnancy, safe communication, health behaviors, obstetric care

## Abstract

**Background:**

In the medical field of obstetrics, communication plays a crucial role, and pregnant women, in particular, can benefit from interventions improving their self-reported communication behavior. Effective communication behavior can be understood as the correct transmission of information without misunderstanding, confusion, or losses. Although effective communication can be trained by patient education, there is limited research testing this systematically with an app-based digital intervention. Thus, little is known about the success of such a digital intervention in the form of a web-app, potential behavioral barriers for engagement, as well as the processes by which such a web-app might improve self-reported communication behavior.

**Objective:**

This study fills this research gap by applying a web-app aiming at improving pregnant women’s communication behavior in clinical care. The goals of this study were to (1) uncover the potential risk factors for early dropout from the web-app and (2) investigate the social-cognitive factors that predict self-reported communication behavior after having used the web-app.

**Methods:**

In this study, 1187 pregnant women were recruited. They all started to use a theory-based web-app focusing on intention, planning, self-efficacy, and outcome expectancy to improve communication behavior. Mechanisms of behavior change as a result of exposure to the web-app were explored using stepwise regression and path analysis. Moreover, determinants of dropout were tested using logistic regression.

**Results:**

We found that dropout was associated with younger age (*P*=.014). Mechanisms of behavior change were consistent with the predictions of the health action process approach. The stepwise regression analysis revealed that action planning was the best predictor for successful behavioral change over the course of the app-based digital intervention (β=.331; *P*<.001). The path analyses proved that self-efficacy beliefs affected the intention to communicate effectively, which in turn, elicited action planning and thereby improved communication behavior (β=.017; comparative fit index=0.994; Tucker–Lewis index=0.971; root mean square error of approximation=0.055).

**Conclusions:**

Our findings can guide the development and improvement of apps addressing communication behavior in the following ways in obstetric care. First, such tools would enable action planning to improve communication behavior, as action planning is the key predictor of behavior change. Second, younger women need more attention to keep them from dropping out. However, future research should build upon the gained insights by conducting similar internet interventions in related fields of clinical care. The focus should be on processes of behavior change and strategies to minimize dropout rates, as well as replicating the findings with patient safety measures.

**Trial Registration:**

ClinicalTrials.gov identifier: NCT03855735; https://classic.clinicaltrials.gov/ct2/show/NCT03855735

## Introduction

### Background

In the dynamic landscape of medical internet research, the pursuit of effective interventions and preventive health programs demands a comprehensive understanding of diverse populations, including pregnant women and their unique needs. This paper unveils the outcomes of formative research and preliminary results within the realm of medical and preventive health, exploring an innovation and technology in terms of a digital intervention, that is, apps aiming at improving communication behavior. Formative research, characterized by its emphasis on gathering insights from the intended beneficiaries, emerges as a fundamental tool for tailoring interventions to meet the unique requirements of diverse communities [1]. By demonstrating the integral role of formative research in the early stages of program development, we aim to provide a compelling case for its incorporation in the toolkit of researchers and experts working in the field of medical internet research. In this paper, we outline the potential of using digital tools like apps for improving communication behavior for patient safety and the risks involved with regard to dropouts in app-based interventions. Lastly, we outline a behavior change theory to model communication behavior, which may help to map out the health-related behavior more systematically and which was used for our research investigating communication behavior and app usage.

### Patient Safety in Health Care

Patient safety is defined as the absence of harm that could have been prevented in patients. For achieving patient safety, health care should be delivered in an optimal manner, trust should be built among all involved individuals, and misunderstanding, information loss, or error occurrence should be prevented [2]. Thus, patient safety requires effective communication behaviors among health care professionals, patients, their partners, or accompanying persons [2]. In particular, in obstetric care, this holds true [2,3] because women in labor have to express their needs and wishes even in the face of stress and barriers to ensure their active role in the obstetric process. Communication behavior can be measured and taught [3-5] and is a reliable approach for improving patient safety [6,7]. Communication behavior involves multiple individuals, including patients, health care workers, and partners [8-13]. This encompasses not only the importance of perceiving a supportive environment that guarantees an open exchange of concerns and potential solutions but also the individual’s competency to communicate safely. Such competency consists of the self-reported communication skills that are based on Rider and Keefer’s [14] competencies and are impacted by determinants of the communication behavior, that is, self-efficacy, intention formation, and planning [15-19].

### Communication Behavior

Communication is defined as a process involving the exchange of cognitions and emotions through verbal and nonverbal actions [20,21]. In this work, we define patients’ communication similarly to communication in health care workers to keep definitions for both groups aligned. Previous work [8,10] performed over the scope of this project has defined communication in line with Rider and Keefer’s definition [14]. They describe a set of skills including the creation and sustainability of a therapeutic relationship, use of effective listening, prompt and effective responding, and effective communication [14]. Effective communication is the correct transmission of information without misunderstanding, confusion, or losses.

Although effective communication has been shown to be of importance in preventing errors in medical care as well as in patient-provider relationships [6,22-24], only few studies have investigated effective communication behavior among those receiving obstetric care. Moreover, there is limited evidence for innovative tools aiming at increasing effective communication among pregnant women and their support networks [8,11,25]. Previous research has mainly investigated face-to-face interventions in clinical care or hospital settings [26,27]. Although traditional face-to-face interventions demonstrate efficacy, they tend to show several disadvantages concerning feasibility, such as higher financial constraints, limited utilization due to mobility constraints, or scheduling and time issues [28-31]. These constraints of traditional face-to-face interventions also call for cost-effective, convenient, instantly available, and scalable alternative solutions. One of these alternatives, successfully implemented across multiple therapeutic areas, including the promotion of health behavior change, is support via the internet, digital interventions, and apps in the medical field [32-35].

### Digital Interventions and Apps

Digital interventions and apps (also called as medical internet support or web-based communication training) have shown several advantages over traditional face-to-face interventions, such as increased ease of accessibility and personalized interactions with real-time feedback. Furthermore, they offer the opportunity for scalability to larger populations, including individuals who live in remote areas. Moreover, such digital interventions can be relatively cost-effective compared to traditional formats [36,37]. Although there is clear general evidence regarding digital interventions, there is scarcity of research on those targeting to foster effective communication.

The same holds true regarding the applicability and integrability of traditional health behavior change theories such as the health action process approach (HAPA) to explain health behavior changes in digital interventions (ie, smartphone apps). Indeed, literature shows how interventions supporting motivational and volitional processes prove effective [8,9,38]. However, the HAPA model has been rarely applied to interventions targeting effective communication [8,9], and it is hardly ever used to explain communication behavior in the context of digital interventions or their dropout of pregnant women. Therefore, we review dropout in more detail.

### Factors Associated With App Usage and Dropout From Digital Interventions

Early dropout from digital interventions is a key problem [39], as the intervention use is discontinued. This needs more attention because if users drop out, which might occur as often as 1 in 2 cases [40], efficacy is limited, and the reach and generalizability of the obtained results are diminished [39]. However, little is known about the factors associated with dropout [39]. Accordingly, more research investigating and identifying such factors is needed, especially in the context of communication behavior and giving birth.

Looking at the general literature on the potential risk factors for early dropout in digital interventions, the following sociodemographic and behavior change factors were identified: age, education, and social support [41,42]. Although there is no previous study on dropout from digital interventions addressing effective communication of pregnant women, evidence from other areas with digital interventions exist. For example, Wu and colleagues [43] investigated dropout in a blended care cognitive behavioral intervention. They highlighted that a higher dropout rate was associated specifically with female gender, poorer financial status, and the absence of a college degree. Additionally, Gao et al [44] found that younger patients and those who were less educated were more likely to drop out from digital intervention studies. Other factors associated with early dropout were marital status (higher probability of divorced individuals to drop out) and ethnicity [45]. Besides the sociodemographic factors, according to Davis and Addis [46], psychological determinants should also be considered while examining dropouts from digital health interventions. According to [47,48], users with low intention to change their behavior have been found to drop out more often from digital interventions. A study by Schroé and colleagues [49] further investigated why users discontinued the use of digital health interventions. Their results highlighted that whereas sociodemographic factors were predictive of early dropout, psychological determinants such as action planning and self-monitoring were associated with completion of digital interventions [49]. This is in line with other research highlighting that self-monitoring [50] and higher intrinsic motivation were associated with lower attrition rates [51]. A theory that could bring the different factors together to enable systematic research is the aforementioned HAPA model, which is described in more detail below.

### HAPA Model to Understand and Improve Behavior Change

Self-reported communication behavior constitutes a preventive health behavior [25] and may be fostered by the same factors and processes that health psychology literature has repeatedly showcased [52-54]. HAPA proved to be a useful theory [11] essentially since it considers the interplay of resources, barriers, as well as the well-known behavior intention gap [54,55]. The HAPA model is divided into 2 distinct phases: (1) the motivational phase in which individuals consider their competencies’ determinants such as self-efficacy, expectations about behavioral outcomes (outcome expectancies) and formulate a behavioral intention (eg, to communicate in the birthing context), and (2) the volitional phase, wherein pregnant women develop and enact behavioral plans in order to bring the intentions to behavioral actions. This whole process is shaped by social-cognitive barriers and facilitators that may originate externally or stem from women’s personal belief, which is also called self-efficacy [54,56]. According to the HAPA model, individuals need to first form an intention, which is based on outcome expectancies and self-efficacy, before acting accordingly. Hence, the pathway of intention on the actual behavior is mediated by action planning [8,11,57]—with action planning being more proximal to behavior, and intention, outcome expectancies, and self-efficacy being more distal to behavior.

In order to improve communication behavior, interventions must be tailored to social-cognitive barriers and facilitators of the target population. Previous evidence has demonstrated that classical face-to-face interventions based on motivational and volitional theories such as HAPA are effective in improving self-reported communication [8,9,38]. It should be noted that most of these findings stem from interventions that were solely offered to health care workers [25], but more attention needs to be paid to patient education. This is the basis of our study with pregnant women randomized into an intervention group or a waitlist control group.

### Goal of This Study

As previously outlined, there is a need for further studies to investigate effective communication behaviors of pregnant women within the context of a digital intervention. The goals of our study were 2-fold. First, we aimed to uncover the potential risk factors for early dropout from a digital intervention. Second, we aimed to investigate the social-cognitive factors that would predict the self-reported communication behavior after having used the digital intervention. Thus, the hypotheses are as follows:

1. Hypothesis 1: Sociodemographic factors play a larger role in predicting dropout during a digital intervention relative to behavior change variables.
2. Hypothesis 2: The social-cognitive factors outlined by HAPA (self-efficacy, outcome expectancy, and action planning) predict self-reported communication behavior in pregnant women over the course of the app-based intervention.
3. Hypothesis 3: More distal HAPA variables (intention, outcome expectancies, and self-efficacy) indirectly relate with self-reported communication behavior mediated by action planning.

## Methods

### TeamBaby Project

This study stems from a larger project named TeamBaby, which was tasked with developing interventions to improve communication behavior between those who receive and provide obstetric care. One of the interventions was a digital intervention, that is, an app (actually, a web-app). Data collected from the TeamBaby web-app were used to investigate our hypotheses. The TeamBaby project was funded by the German Innovation Fund (project 01VSF18023) of the Gemeinsamer Bundesausschuss and preregistered (ClinicalTrials.gov identifier: NCT03855735) on February 27, 2019.

### Recruitment and Procedures

#### Ethics Approval

Ethics approval for data collection in the maternity clinics was granted by the ethics committee for human research of the University Hospital Ulm (114/19) and the ethics committee for medical research of the University Hospital Frankfurt am Main (19-292). Informed consent was provided in the registration process, and all data were anonymized by providing users with a random ID that could not be linked to user emails or personal IDs. No compensation was provided for participation in this study.

#### Participants

**Figure 1:**
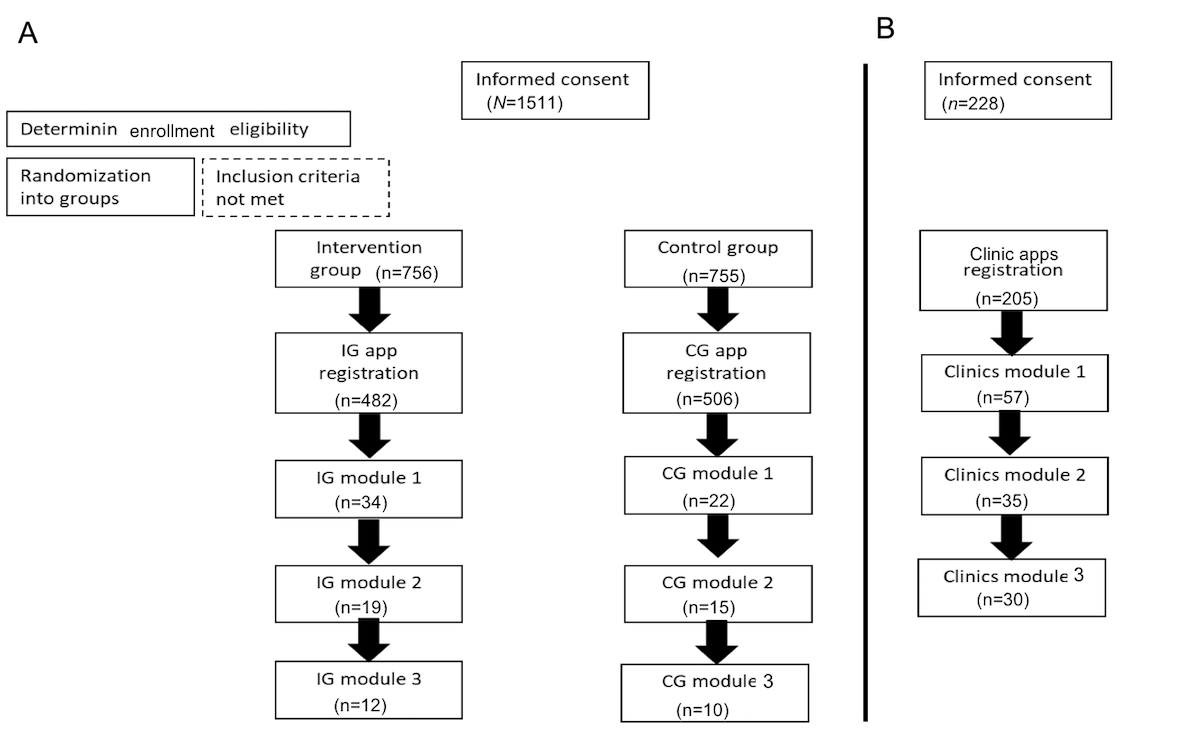
Flowchart of the study participants. (A) Study participants using the app as intervention group versus being randomized to the control group and using the app only 2 weeks later. (B) Study flow for the clinic’s intervention group. CG: control group; IG: intervention group.

Participants were recruited into this study, as outlined in Figure 1. Participants represented a pragmatic sample. Sample calculations were performed prior to data collection for an assumed dropout of 20%. We estimated that 176 or more individuals would be needed to recruit [9]. All recruited women were able to register to use the TeamBaby web-app if they were residing in Germany, either during the time of our study (if randomized into the intervention group) or 2 weeks later (if randomized into the waitlist-control arm), that is, pregnant women and their support persons.

Pregnant women were recruited through 1 of the 2 recruitment channels (Figure 1): (1) those who sought treatment during pregnancy in project-affiliated clinics or (2) those who were currently pregnant and based anywhere in Germany. With respect to the former group, project members worked together with obstetricians to recruit patients. The Germany-wide recruitment utilized social media and targeted advertising to promote this study. In addition, flyers were placed in health care clinics and pharmacies across the country. Women were eligible to participate if they were currently pregnant, had sufficient knowledge of German, and were at least 18 years old.

Participants were recruited into this study upon completing a web-based questionnaire. Women recruited in the clinics were invited to register with the web-app. Women from the Germany-wide recruitment were randomized into either a treatment group or waitlist-control group. The treatment group was presented with a link to use the web-app directly. The waitlist-control arm was provided with a link to the web-app 2 weeks later. All users of the web-app were presented with a series of questions at regular timepoints to determine whether there were changes in HAPA variables and communication behavior as users progressed through the 3 modules of the web-app. Participants who completed less than 2 modules were considered as early dropouts.

#### Intervention Content

The TeamBaby web-app provided guidance on how to work effectively with health care workers. The web-app consisted of 10 lessons, wrapped in 3 modules, which were developed and structured based on the processes of behavior change as set out by the HAPA model. The first set of lessons was designed to increase the outcome expectations of effective communication behavior and create an intention to adopt self-reported communication practices. The subsequent lessons were designed to increase the belief or trust in oneself to employ effective communication and enable users to make tangible plans for implementing self-reported communication behavior. More detailed information about the modules and the content can be found in Multimedia Appendix 1.

#### Measures

Participants were asked to complete questions relating to communication behavior and HAPA variables at 4 timepoints: before starting the first module and after completing each subsequent module. The assessment of self-reported communication behavior was based on Rider and Keefer’s competencies [14] and adapted to address pregnant women’s behavior in previous publications [12]. As the aim of the research was to understand the underlying social-cognitive processes of communication, the items were developed to capture self-reported communication behavior. Table 1 presents the items [14,58,59] used to evaluate each variable.

**Table 1:** Measured health action process approach and self-reported communication variables.

Variables	Item example	Range^a^
Communication behavior (7 items) [14]	During pregnancy, I always have communicated my needs clearly.	1-6
Outcome expectancy (single item) [58]	If I communicate well with doctors and midwives, my preferences can be considered during childbirth.	1-6
Intention (single item) [59]	I intend to always make sure that I communicate effectively with the doctors and midwives.	1-6
Self-efficacy (single item) [59]	I am sure I can communicate well even when I am tired or exhausted.	1-6
Action planning (single item) [58]	I have planned precisely how to communicate well while giving birth.	1-6

^a^Range of 1-6 spans from “does not apply at all” to “does apply fully.”

### Sociodemographic Data

In addition to behavior change measures, demographic information was collected. Participants reported their age, marital status, highest level of education, and nationality.

### Aggregated Variables

Participant communication scores were combined into a single item for each individual by taking the average across the different communication behaviors. This was expressed as overall communication. The implicit assumption here is that more effective communication within the described obstetric setting should facilitate a safer birth.

### Statistical Analysis

All analyses were conducted using the R [60] and RStudio [61] software. Significance was determined at the 5% level. The aim was to determine what variables would predict early dropout from the web-app. Early dropout was expressed as a binary variable: participants were marked as dropping out early if less than 2 modules were completed. For example, participants who completed 2 or 3 modules were marked as 0 (ie, not dropped out early), while participants who completed only 1 module or none were marked as 1 (ie, dropped out early).

To investigate hypothesis 1, a general logistic regression model using the glm function was built to identify whether HAPA variables, age, marital status, education, and recruitment channel predicted dropout. Recruitment channel was a categorical variable that reflected entry into the app. Participants entered the app either directly through clinical recruitment or in the Germany-wide recruitment after randomization into the intervention group or after the waiting time when being randomized into the waitlist control arm. In the logistic regression model, the clinical recruitment group was the reference group to which the other recruitment channels were compared to.

A hierarchical regression was performed to investigate hypothesis 2, using the “lm” function available in Base R; HAPA variables were sequentially added to build a final model that predicts post web-app communication. Table 2 outlines how the predictor and outcome variables were operationalized in the model. Following the construction of a model to explain changes in post web-app communication, possible processes for behavior change were proposed. To investigate hypothesis 3, a structural equation model using the lavaan package [62] was built to identify how HAPA variables related to one another and in turn contributed to changes in post web-app communication.

**Table 2:** Overview of the operationalization of social-cognitive predictors and communication behavior.

Type	Variable	Operationalization	Example
Outcome	Post web-app communication, expressed as C_t_	Respondent’s most recent overall communication score after having completed at least 1 module of the web-app. Only individuals who completed at least 1 module were included in the analyses to ensure the data captured those that used the web-app.	If an individual had communication scores after modules 1, 2, and 3, only the response after module 3 was used.
Predictor	Outcome expectancy at the preceding timepoint, expressed as OE_t-1_ Intention at the preceding timepoint, expressed as Intention_t-1_ Self-efficacy at the preceding timepoint, expressed as SE_t-1_ Action planning at the preceding timepoint, expressed as AP_t-1_	A respondent’s HAPA^a^ variable score at the timepoint preceding the last available communication score	If an individual had a communication score after module 3, their HAPA variable scores after module 2 were used for the predictor variables.

^a^HAPA: health action process approach.

Multimedia Appendix 2 outlines the intercorrelation between all used social-cognitive HAPA determinants as well as self-reported communication behavior over the course of the app-based intervention. Since the abovementioned analysis includes as many timepoints as possible, all variables at the different timepoints were included.

## Results

### Study Participants

Overall, 1187 women were recruited into this study, of which 988 were from the Germany-wide recruitment (Figure 1A). Of those in the Germany-wide sample, 506 were randomized into the waitlist control arm (control group app registration), and 482 were randomized into the intervention arm (intervention group app registration). In the clinics, 199 pregnant women were recruited (after the registration of 205 women with the app). The majority of the participants were aged 30-39 years (n=881), had a higher education status (n=763), and were married (n=759).

### Descriptive Statistics

Sociodemographic data are depicted in detail in Table 3 below.

**Table 3:** Sociodemographic characteristics of expectant mothers (N=1187).

Characteristics			Values, n (%)
**Age^a^ (years)**			
	18-29	159 (14.23)	
	30-39	881 (78.87)	
	40-49	77 (6.89)	
**Marital status^b^**			
	Single	31 (2.67)	
	In a committed relationship	366 (31.5)	
	Married/registered partnership	759 (65.32)	
	Divorced/separated/widowed	6 (0.51)	
**Highest educational level^c^**			
	No school-leaving qualification	21 (1.81)	
	Secondary or elementary school leaving	78 (6.74)	
	Secondary school diploma	137 (11.83)	
	A-levels	763 (65.89)	
	Completed vocational training	25 (2.16)	
	University degree^d^	34 (2.94)	
	University degree^e^	100 (8.64)	

^a^76 missing values for age.

^b^31 missing values for marital status.

^c^35 missing values for highest educational level.

^d^Special German university degree (Hochschule).

^e^University degree.

### Predicting Dropout

Of the 1187 pregnant women who were recruited and started using the web-app, 1124 dropped out of the intervention, as indicated by completion of less than 2 modules. A general logistic model was estimated to investigate hypothesis 1 and to determine whether social-cognitive HAPA variables and communication behavior as well as sociodemographic characteristics might predict early dropout (completing less than 2 modules). Thereby, the predictive capacity of 4 HAPA variables and behavior along with age, education, and marital status was tested: intention, outcome expectancy, self-efficacy, and action planning, as well as sex, education, and marital status. As Table 4 highlights, only age was a significant predictor of early dropout. In other words, younger pregnant women were more likely to drop out from the digital intervention at an earlier stage. Accordingly, hypothesis 1 can be empirically supported.

**Table 4:** Parameter table of the generalized linear model predicting early dropout from health action process approach variables and sociodemographic characteristics.

Variable	Estimate (SE)	*t* test *(df)*^a^	*P* value
Outcome expectancy_tt1_^b^	.005 (.164)	0.034 (418)	.97
Intention_tt1_	–.254 (.153)	–1.658 (418)	.09
Self-efficacy_tt1_	.184 (.121)	1.516 (418)	.13
Action planning_t1_^c^	.204 (.112)	1.816 (418)	.07
Age	–.094 (.038)	–2.469 (418)	.014^d^
Education	–.178 (.161)	–1.106 (418)	.27
Marital status	–.363 (.302)	–1.200 (418)	.23
Recruitment channel=Germany-wide recruitment, randomized into the intervention group (compared to clinical recruitment)	.880 (.523)	1.683 (418)	.09
Recruitment channel=Germany-wide recruitment, randomized into the waitlist control arm (compared to clinical recruitment)	.374 (.540)	0.692 (418)	.49

^a^2-sided *t* test.

^b^tt1: measurement after completing module 1.

^c^t1: module 1 (lessons 1-3).

^d^β is significant at *P*=.05; *R*²=0.13.

### Predictors of Self-Reported Communication Behavior

Hypothesis 2 tests whether socio-demographic variables and social-cognitive HAPA variables (self-efficacy, outcome expectancy, and action planning) would predict self-reported communication behavior in pregnant women over the course of the app-based intervention (see Table 5 for details). In the first series of models, each sociodemographic variable was added in a stepwise fashion to predict communication behavior. Adding age (*F*_1,93_=1.16; *P*=.28), education (*F*_1,92_=0.12; *P*=.66), and family status (*F*_1,91_=0.39; *P*=.54) did not significantly improve the prediction of communication behavior scores. In the subsequent models, HAPA variables were added (Table 5), and intention was added. Upon inclusion, most HAPA variables improved the model fit. For the motivational phase of the HAPA model, outcome expectancy (*F*_1,90_=4.88; *P*=.03) and intention (*F*_1,98_=8.65; *P*=.004) improved the model fit, while task self-efficacy (*F*_1,88_=2.11; *P*=.15) did not improve the model fit. For the volitional phase, action planning (*F*_1,87_=17.74; *P*<.001) improved the prediction of communication scores.

After including all the variables of HAPA along with sociodemographic variables into the model (for model comparisons, see Table 5), only action planning (β=.331; *P*<.001) significantly predicted communication behavior (Table 6 for further parameter estimates). Accordingly, hypothesis 2 could be partially empirically supported.

**Table 5:** Hierarchical regression model comparison of sociodemographic and social-cognitive health action process approach variables predicting communication behavior.

Model name	Comparison model	Predictors	*F* test *(df)*	*P* value
Age	Null	Age	2.165 (1, 92)	.15
Education status	Age	Age + education	0.045 (1, 91)	.83
Marital status	Education status	Age + education + marital status	0.365 (1, 90)	.55
Outcome expectancy	N/A^a^	Outcome expectancy	4.430 (1, 89)	.04^b^
Intention	Outcome expectancy	Outcome expectancy + intention	8.457 (1, 88)	.005^c^
Self-efficacy	Intention	N/A	1.955 (1, 87)	.17
Action planning	Self-efficacy	N/A	17.68 (1, 86)	<.001^d^

^a^N/A: not applicable.

^b^β is significant at *P*=.05.

^c^β is significant at *P*=.01.

^d^β is significant at *P*=.001.

**Table 6:** Parameter table of hierarchical regression model predicting communication behavior.

Variable	Estimate (95% CI)	SE	*P* value
Age	–. 016 (–.051 to .018)	.02	.34
Education status	–. 047 (–.154 to .060)	.053	.39
Marital status	–. 100 (–.442 to .243)	.172	.57
Outcome expectancy	.14 (–.047 to .336)	.096	.14
Intention	.046 (–.143 to .236)	.095	.63
Self-efficacy	.007 (–.136 to .149)	.072	.93
Action planning	.305^a^ (–.161 to .449)	.072	<.001

^a^β is significant at *P*=.001.

### Mediation Model

**Figure 2:**
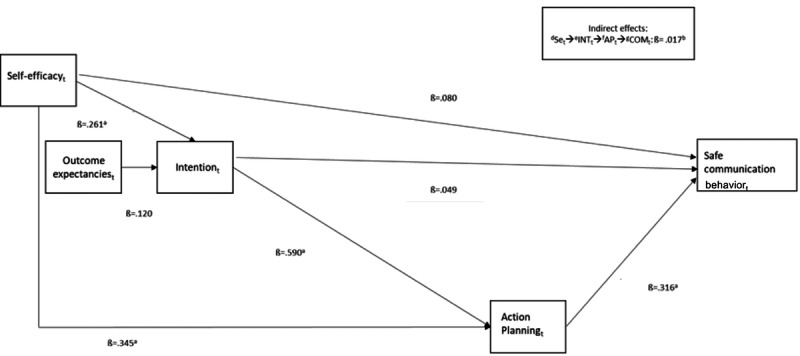
Regression model of social-cognitive health action process approach variables and safe communication behavior across all groups. Taken together, communication behavior is significantly predicted by action planning, and action planning mediates the impact of self-efficacy and intention on self-reported communication behavior with β=.017 (comparative fit index=0.994; Tucker–Lewis index=0.971; root mean square error of approximation=0.055). AP: action planning; COM: safe communication behavior; INT: intention; SE: self-efficacy; t: reflects a relative timepoint.

To test hypothesis 3 and thus whether distal HAPA variables (intention, outcome expectancies, self-efficacy) are mediated through planning, a path model was facilitated (Figure 2 and Table 7). Indeed, a sequential mediation emerged from self-efficacy to intention to action planning to self-reported communication behavior. This likewise entailed the indirect mediation from intention via action planning to self-reported communication behavior. Conversely, no serial mediation was found from outcome expectancies to intention via action planning to self-reported communication behavior. Accordingly, hypothesis 3 could only be empirically supported regarding 2 of the 3 distal HAPA variables, namely, self-efficacy and intention.

**Table 7:** Results of the path model depicted in Figure 2.

Predictor variable	Outcome variable	Estimate (95% CI)	SE	*P* value
Self-efficacy	Intention	.353^a^ (.152 to .555)	.103	.001
Outcome expectancy	Intention	.105 (–.108 to .318)	.109	.33
Intention	Action planning	.408^a^ (.242 to .574)	.085	<.001
Self-efficacy	Planning	.322^a^ (.148 to .497)	.089	<.001
Planning	Communication behavior	.471^a^ (.311 to .631)	.082	<.001
Intention	Communication behavior	.053 (–.163 to .270)	.111	.63
Self-efficacy	Communication behavior	.108 (–.069 to .285)	.090	.23

^a^β is significant at *P*=.001.

## Discussion

### Principal Findings

In this study, we investigated the determinants of dropout from an app-based intervention for pregnant women and the mechanisms of adopting self-reported communication behavior. Regarding both aspects, variables from HAPA were measured and evaluated with respect to their predictive capability. Consistent with hypothesis 1, dropout analyses found that only age was predictive, and none of the HAPA variables played a role. Communication behavior was only predicted significantly by one of the HAPA variables, namely, action planning. A serial mediation emerged from intention to self-reported communication behavior via action planning. In detail, communication behavior was significantly predicted by action planning, and action planning mediated the impact of self-efficacy and intention on self-reported communication behavior. These findings match previous findings that identified age as a predictor of dropout from digital interventions [41,42]. However, in contrast to the aforementioned studies, no other sociodemographic predictors emerged. Moreover, this study deviates from previous dropout investigations, as no HAPA variables predicted early dropout. This might be a result of differences in the target group or context of the digital tool between studies; predictors of dropout from digital interventions might depend on aspects of specific intervention types and could vary based on the timepoint of dropout, as revealed in a previous research [63]. Regarding the latter, it should be noted that our study only investigated early dropout.

With respect to mechanisms of adopting self-reported communication behavior, the results were in line with hypotheses 2 and 3: the data demonstrated that HAPA variables predicted the self-reported communication behavior. Whether this is a result of the behavior change context (ie, the app) still needs to be determined. However, the findings are in line with previous evidence that HAPA assumptions match the data and accordingly are able to explain the changes in self-reported communication behavior [8,10].

Our findings of early dropout from the app-based intervention are partially in line with those of other studies, in which younger participants were more likely to drop out from digital interventions [42,64-66]. The relationship between age and dropout could be a result of higher perceived need among older women, that is, older women, through more life experiences and previous pregnancies, may realize a greater need for communication interventions and in turn adhere to the app.

Behavior change variables did not predict dropout from the app-based intervention. This is in contrast to the results of previous studies that have shown that behavior change variables are associated with dropout [63]. Among others, this study shows outcome expectancies as a crucial predictor of retention in digital interventions and likewise concludes that the perception of unmet needs and expectations might be a determinant of dropout [63]. In the context of self-reported communication behavior, expectant mothers’ outcome expectancies focused on safe child delivery instead of self-reported communication behavior and the accommodation of individual preferences as the item wording suggests. An alternative explanation would be that the relationship between behavior change variables and dropout is context-specific, as a previous study implicates [63]. In any case, our study shows the determinants of dropout from an app-based intervention. This is an important insight for patient safety interventions in obstetrics because it can be used by future tools to prevent early dropout and maximize the amount of support that pregnant women receive. However, future research should further investigate the contextual variability of predictors of early dropout in digital intervention studies. This is particularly important because app-based interventions generally show high dropout rates [67-70].

Previous studies [8,11] have shown that social-cognitive variables are associated with pregnant women’s safe communication behavior in general. Our study demonstrates that some of these associations also drive change in self-reported communication behavior during a digital intervention. This is of importance for theoretical understanding and practitioners; it shows how apps might elicit and affect changes in self-reported communication behavior, highlighting pathways which future interventions can focus on and improve its effectiveness. This might be useful for designing future apps in the specific field of pregnancy and giving birth.

It is striking that not all associations in HAPA emerged as theorized. First, it became apparent that action planning was the single best predictor of change in self-reported communication behavior. Although the predictive capacity of action planning is expected from its association with behavior within the HAPA model, it was not hypothesized that action planning would emerge as the sole predictor of behavior change. The reason could be pregnant women participating in the app were taught to think of how and when they might communicate effectively, that is, making concrete action plans, which worked well in the app, while other variables were not addressed as effectively. In addition, action planning was targeted in the last lesson of the app, which might have resulted in stronger effects due to a shorter time lag and recency effect.

Relating to the process by which pregnant women improve their communication behavior in clinical care, it is likewise striking why only 2 of the indirect effects specified in the HAPA framework emerged. First, there was an indirect effect of self-efficacy on self-reported communication behavior via intention and subsequently via action planning. Second, self-efficacy also directly impacted action planning and thereby indirectly impacted self-reported communication behavior. Notably, the same predictive capacity in explaining self-reported communication behavior in this study replicated findings from a cross-sectional research [8,11]. Indeed, the HAPA model seems to be applicable for predicting several kinds of behavior change in digitally supported interventions like the app used in this study and has shown similar findings overall [71-73].

In a previous randomized trial in patients with insomnia, both action planning and coping planning in the HAPA model were shown to be effective mediators in improving sleep hygiene [71]. In another randomized trial testing a digital tool to promote active lifestyles in patients with type 2 diabetes, the intervention group showed a significant intervention effect for action planning, whereas the control group exhibited a significant effect for coping planning and self-efficacy [72]. Lastly, in an earlier study primarily focusing on reducing salt intake to prevent high blood pressure, both intention and outcome expectancies as well as risk perception were found to be improved by the digital intervention [73].

In the future, digital and nondigital face-to-face interventions should be compared, especially when aiming to improve self-reported communication behavior in obstetrics and preventing dropout [10]. Different app modes showed various degrees of effectiveness in a study on depression [74]. A meta-analytic review [74] showed that apps in combination with personal contact with a therapist are more effective than self-help apps. However, no differences were found between smartphone-based apps and computer- and internet-based interventions. Similarly, there seems to be no difference between human-guided digital interventions and face-to-face psychotherapy [74]. In other areas of research, gamifications in apps have proven to be beneficial [68]. Among other findings, feedback, leaderboards (participants can compare their own progress with that of others), and storytelling (context within the app to create an alternate reality and guide the user) have been shown to be advantageous for digital interventions [68-70]. Those findings provide some guidance for future teams aiming at developing apps and internet interventions in this field.

Findings from this body of research set the stage for iterating on existing apps in clinical care and for developing new apps. However, it seems questionable, what kind of intervention might be sufficient or helpful for those participants at risk for dropping out. On the one hand, flexible digital tools, which allow an automatic dynamic change of modules and learning intensity, might be helpful. On the other hand, an overload of information or special attention to these participants might make them even more prone to drop out. To conclude, future research should further uncover the reasons for dropout of such participants, so that optimal strategies for prevention can be devised.

Given that younger patients are at increased risk of discontinuing the digital intervention at an early stage, it is crucial to make the underlying behavior visible and targetable. We made the first attempt to explain this phenomenon. In the literature, the possibility of using behavior change theories to predict dropout behavior has been demonstrated [63]. Future research should be conducted using different intervention modes as well as different digital incentives (eg, optimal level of gamification, possibility to exchange with other users vs personal contact with midwives, doctors, or other birthing professionals, or more intensive self-help vs person-guided self-help).

### Limitations and Suggestions for Future Research

This study, as a formative research and with preliminary results, has certain limitations with regard to the conclusions drawn from the results. First, there is a possibility that confounding external factors could have been at play during the course of the internet intervention, such as physiological or mental health risk factors. Second, bias and self-selection might have confounded the web-app data in the sense that only certain women volunteered to participate and continue the app-based intervention. Future internet intervention studies should try to recruit a more diverse sample and find concrete reasons for them completing the app or dropping out. It seems possible that both flexible feasibility questions in the context of the app as well as effectiveness ratings and satisfaction ratings could provide more information about usage behavior.

For the time frame between intervention start and the last timepoint, we have conducted a test of factors predicting dropout, from which we concluded that only younger age at intervention start predicted early dropout. However, factors associated with the selection to participate in the intervention could not be uncovered in our presented design. Future research should target a wider age range of pregnant women to gain further insight between age groups and dropout via subgroup analyses. With a larger sample, it also seems possible to examine the age categories and dropout behavior in more detail.

As mentioned previously, our data do not allow conclusions regarding the motivations of dropout and study retainment—a topic that future studies should investigate further. Relatedly, it would have been important to have more finely spaced time intervals for measurement points, which could add valuable information on the interplay of the processes underlying behavioral change and the topics of the particular lessons that were covered. Additionally, it should be acknowledged that the scales used to measure the social-cognitive variables of HAPA were not previous validated in German language based on evaluating the communication behavior. Hence, the measurement qualities of the scales in the German population might be limited.

The main contribution of this study can be seen in that it is the first attempt of employing a digitally enhanced internet intervention aiming at fostering self-reported communication behavior within a clinical sample in the context of obstetric care. This has innovation potential, as it shows that technology in terms of a digital intervention, that is, apps aiming at improving communication behavior can make a difference. Therefore, this study sheds light on the mechanisms underlying self-reported communication behavior and its improvement while also investigating the potential predictors of dropout in an app-based intervention. This contribution yields both practical and theoretical implications. On a theoretical note, our study contributes to a deeper understanding of the genesis of self-reported communication behavior, thereby highlighting various points of the psychological processes that future interventions could address, such as action planning and self-efficacy. Likewise, practical implications arise as our study presents an initial framework for improving effective communication via the app in a clinical sample and explores how to maximize its effectiveness by retaining participants at risk of early dropout. The variables of the HAPA model can function as a toolkit, with a particular focus on action planning, self-efficacy beliefs, intention to communicate effectively, and app users’ age.

## Appendix

Multimedia Appendix 1 Specifications of the TeamBaby web-app.

Multimedia Appendix 2 Intercorrelation between all used social-cognitive determinants as well as self-reported communication behavior over the course of the app-based intervention.

## Abbreviations

HAPA: health action process approach

## Data Availability

The data that support the findings of this study are available on request from the corresponding author SL. The data are not allowed to be made publicly available due to privacy and data security reasons of the research participants.
