# Supplementary material for: Effective Communication Supported by an App for Pregnant Women: Quantitative Longitudinal Study
Source: JMIR Hum Factors. 2024 Apr 26;11:e48218. doi: 10.2196/48218 (PMC11087862; doi:10.2196/48218)
Supplement: Multimedia Appendix 1 [file humanfactors_v11i1e48218_app1.docx]

**Multimedia Appendix 1**

Specifications of the TeamBaby web-app

|  | **Details** | **Further information** |
| --- | --- | --- |
| Purpose | Facilitate effective communication between those receiving and providing obstetrics care. | Targeting pregnant women |
| Target group | Pregnant women and their partners | Partners were not included in this study due to low recruitment numbers |
| Length | Mean of 2.00 hours | Range: 53 minutes to 302 minutes (SD = 0.51) |
| Active period | February 2021 to June 2022 | An improved version of the app is online available: https://teambaby.health |
| Modules | Preparation for lessons | Participants complete self-reflection questionnaire to identify needs and preferences of birthing. |
| Lesson 1 | Communication competencies | Introduction and explanation of effective communication (behaviour) with multiple choice. |
| Lesson 2 | 4 sides of communication | This exercise is about reducing the chances of a misunderstanding with the communication partner. Situation examples are provided. Answer format: open questions and multiple-choice answers. |
| Lesson 3 | Speaking up | Practical lesson with the goal of expressing own wishes, as soon as their needs are at risk. Answer format: Multiple choice and open questions with typical situations during the birth process. |
| Lesson 4 | Closed-loop | Practical lesson - how to avoid misunderstandings with the “close-the-loop” strategy.  The main goal is, how to ask specific questions when something is unclear. Answer format: multiple choice with an example communication process between doctor and mother to be as well as self-reflection questions. |
| Lesson 5 | Empathy and change of perspective | Lesson about the emotional side of communication. Empathy is crucial to being well supported during childbirth. Putting yourself in the shoes of midwives, doctors and support persons in terms of feelings, fears, concerns and tasks. Answer format: open questions. |
| Lesson 6 | Communication strategy ISBAR | Lesson with an exercise how to communicate concerns quickly and effectively. One way to do this is to learn how to effectively structure content. Answer format: open questions and multiple choice guided by an example situation. |
| Lesson 7 | 5-point preparation | Lesson concerning best preparation for conversations and appointments with medical professionals. The goal is a good preparation for an effective doctor-patient relationship. Answer format: open questions considering the presented 5-point preparation. |
| Lesson 8 | Active stress management | Lesson - how to deal with stressful situations (even after giving birth) and what tricks of the trade can be used. Answer format: multiple choice considering an example situation as well as example dialogue between expectant mother and support person. |
| Lesson 9 | (Behavioural) action planning | Lesson in which participants develop a plan to practice and apply tools and strategies to communicate effectively. |
| Lesson 10 | Repetition | Repetition of Lesson 1-9 with multiple choice as well as rating questions concerning the own competencies. |
