# Supplementary material for: Effective Communication Supported by an App for Pregnant Women: Quantitative Longitudinal Study
Source: JMIR Hum Factors. 2024 Apr 26;11:e48218. doi: 10.2196/48218 (PMC11087862; doi:10.2196/48218)
Supplement: Multimedia Appendix 2 [file humanfactors_v11i1e48218_app2.docx]

**Multimedia Appendix 2.** Intercorrelation of studied variables.

|  |  | 1 | 2 | 3 | 4 | 5 | 6 | 7 | 8 | 9 | 10 | 11 | 12 | 13 | 14 | 15 | 16 | 17 | 18 | 19 | 20 |
| --- | --- | --- | --- | --- | --- | --- | --- | --- | --- | --- | --- | --- | --- | --- | --- | --- | --- | --- | --- | --- | --- |
| 1. OEc T1 | r | 1 |  |  |  |  |  |  |  |  |  |  |  |  |  |  |  |  |  |  |  |
|  | P |  |  |  |  |  |  |  |  |  |  |  |  |  |  |  |  |  |  |  |  |
| 2. OE T2 | r | .555a | 1 |  |  |  |  |  |  |  |  |  |  |  |  |  |  |  |  |  |  |
|  | P | <.001 |  |  |  |  |  |  |  |  |  |  |  |  |  |  |  |  |  |  |  |
| 3. OE T3 | r | .549a | .837a | 1 |  |  |  |  |  |  |  |  |  |  |  |  |  |  |  |  |  |
|  | P | <.001 | <.001 |  |  |  |  |  |  |  |  |  |  |  |  |  |  |  |  |  |  |
| 4. OE T4 | r | .431a | .673a | .812a | 1 |  |  |  |  |  |  |  |  |  |  |  |  |  |  |  |  |
|  | P | .001 | <.001 | <.001 |  |  |  |  |  |  |  |  |  |  |  |  |  |  |  |  |  |
| 5. SEd T1 | r | .254a | .241a | .351a | .341a | 1 |  |  |  |  |  |  |  |  |  |  |  |  |  |  |  |
|  | P | <.001 | .006 | .002 | .007 |  |  |  |  |  |  |  |  |  |  |  |  |  |  |  |  |
| 6. SE T2 | r | .219b | .288a | .369a | .332a | .698a | 1 |  |  |  |  |  |  |  |  |  |  |  |  |  |  |
|  | P | .014 | .001 | .001 | .009 | <.001 |  |  |  |  |  |  |  |  |  |  |  |  |  |  |  |
| 7. SE T3 | r | .219 | .274b | .410a | .360a | .562a | .725a | 1 |  |  |  |  |  |  |  |  |  |  |  |  |  |
|  | P | .056 | .017 | <.001 | .004 | <.001 | <.001 |  |  |  |  |  |  |  |  |  |  |  |  |  |  |
| 8. SE T4 | r | .349a | .422a | .552a | .476a | .655a | .617a | .774a | 1 |  |  |  |  |  |  |  |  |  |  |  |  |
|  | P | .006 | .001 | <.001 | <.001 | <.001 | <.001 | <.001 |  |  |  |  |  |  |  |  |  |  |  |  |  |
| 9. INe T1 | r | .319a | .259a | .351a | .539a | .369a | .355a | .437a | .443a | 1 |  |  |  |  |  |  |  |  |  |  |  |
|  | P | <.001 | .003 | .002 | <.001 | <.001 | <.001 | <.001 | <.001 |  |  |  |  |  |  |  |  |  |  |  |  |
| 10. INT2 | r | .223b | .332a | .413a | .529a | .246a | .432a | .421a | .507a | .601a | 1 |  |  |  |  |  |  |  |  |  |  |
|  | P | .011 | <.001 | <.001 | <.001 | .005 | <.001 | <.001 | <.001 | <.001 |  |  |  |  |  |  |  |  |  |  |  |
| 11. INT3 | r | .263b | .398a | .458a | .533a | .267b | .331a | .422a | .548a | .532b | .651a | 1 |  |  |  |  |  |  |  |  |  |
|  | P | .022 | <.001 | <.001 | <.001 | .021 | .004 | <.001 | <.001 | <.001 | <.001 |  |  |  |  |  |  |  |  |  |  |
| 12. IN T4 | r | .292b | .511a | .600a | .558a | .211 | .173 | .329b | .441a | .504a | .565a | .693a | 1 |  |  |  |  |  |  |  |  |
|  | P | .023 | <.001 | <.001 | <.001 | .106 | .191 | .010 | <.001 | <.001 | <.001 | <.001 |  |  |  |  |  |  |  |  |  |
| 13. APf T1 | r | .091b | .065 | .023 | .029 | .355a | .226b | .197 | .378a | .392a | .277a | .269b | .214 | 1 |  |  |  |  |  |  |  |
|  | P | .041 | .476 | .843 | .827 | <.001 | .013 | .092 | .003 | <.001 | .002 | .022 | .104 |  |  |  |  |  |  |  |  |
| 14. AP T2 | r | .137 | .155 | .150 | .208 | .366a | .399a | .433a | .509a | .392a | .416a | .466a | .389a | .627a | 1 |  |  |  |  |  |  |
|  | P | .135 | .085 | .209 | .121 | <.001 | <.001 | <.001 | <.001 | <.001 | <.001 | <.001 | <.001 | <.001 |  |  |  |  |  |  |  |
| 15. AP T3 | r | .255b | .225 | .127 | .185 | .244b | .268b | .341a | .464a | .270b | .377a | .493a | .351a | .616a | .776a | 1 |  |  |  |  |  |
|  | P | .027 | .055 | .271 | .157 | .038 | .023 | .003 | <.001 | .020 | .001 | <.001 | .006 | <.001 | <.001 |  |  |  |  |  |  |
| 16. AP T4 | r | .414b* | .193 | .207 | .198 | .302b | .259b | .445a | .519a | .211 | .283b | .464a | .441a | .549a | .605a | .751a | 1 |  |  |  |  |
|  | P | .001 | .144 | .113 | .124 | .019 | .048 | <.001 | <.001 | .109 | .030 | <.001 | <.001 | <.001 | <.001 | <.001 |  |  |  |  |  |
| 17. COg T1 | r | .077 | .142 | .083 | .111 | .213a | .177 | .097 | .109 | .374a | .249a | .118 | .145 | .312a | .222b | .185 | .155 | 1 |  |  |  |
|  | P | .076 | .115 | .472 | .399 | .000 | .052 | .406 | .409 | <.001 | .005 | .314 | .272 | .000 | .016 | .114 | .241 |  |  |  |  |
| 18. CO T2 | r | .108 | .190b | .060 | .021 | .098 | .242a | .153 | .162 | .333a | .351a | .255b | .143 | .401a | .468a | .447a | .367a | .518a | 1 |  |  |
|  | P | .221 | .030 | .592 | .869 | .268 | .006 | .179 | .208 | <.001 | <.001 | .024 | .273 | <.001 | <.001 | <.001 | .004 | <.001 |  |  |  |
| 19. CO T3 | r | .200 | .176 | .146 | .102 | .061 | .227b | .327a | .372a | .297a | .277b | .424a | .227 | .395a | .547a | .516a | .564a | .475a | .846a | 1 |  |
|  | P | .078 | .126 | .200 | .430 | .601 | .049 | .003 | .003 | .009 | .015 | <.001 | .079 | <.001 | <.001 | <.001 | <.001 | <.001 | <.001 |  |  |
| 20. CO T4 | r | .209 | .157 | .110 | .158 | .084 | .182 | .321b | .352a | .179 | .364a | .398a | .216 | .353a | .512a | .585a | .553a | .345a | .781a | .824a | 1 |
|  | P | .115 | .236 | .406 | .231 | .528 | .172 | .014 | .006 | .184 | .005 | .002 | .103 | .007 | <.001 | <.001 | <.001 | <.001 | <.001 | <.001 |  |

^a^Correlation is significant at .001 level.

^b^Correlation is significant at .01 level.

^c^OE: outcome expectancy

^d^SE: self-efficacy.

^e^IN: intention.

^f^AP: action planning.

^g^CO: self-reported communication behavior.
